# Acute cerebral infarction following a *Trimeresurus stejnegeri* snakebite: A case report

**DOI:** 10.1097/MD.0000000000015684

**Published:** 2019-06-07

**Authors:** Xiangxia Zeng, Jinlun Hu, Xiaohuan Liang, Yixia Wu, Mei Yan, Menghuan Zhu, Yue Fu

**Affiliations:** Department of General Medicine, The First People's Hospital of Foshan (The Affiliated Foshan Hospital of Sun Yat-sen University), Foshan, Guangdong, China.

**Keywords:** acute cerebral infarction, snake bite, thrombosis

## Abstract

**Rationale:**

Acute cerebral infarction after snake bites is rare. The underlying mechanism causing the thrombotic process remains complex and unknown.

**Patient concerns:**

We herein describe a 49-year-old female who was bitten by a *Trimeresurus stejnegeri*. After 4 days of biting, she developed acute ischemic infarct.

**Diagnosis:**

The patient exhibited right side weakness and speech disturbances. Brain computed tomography (CT) scan showed no sign about cerebral hemorrhage symptoms, and brain magnetic resonance imaging (MRI) showed acute ischemic infarct in the left territory. The patient confirmed a diagnosis of acute cerebral infarction following a *T. stejnegeri* bite.

**Interventions:**

The patient received an injection of polyvalent anti-snake venom serum, neuroprotective therapy, and anti-platelet aggregate treatment.

**Outcomes:**

At the 3-month follow-up visit, the patient's left lower extremity swelling disappeared, the right limb muscle strength recovered, and the modified Rankin scale (mRS) score was 4 points.

**Lessons:**

The patient was diagnosed with acute ischemic infarct interrelated to snake bite; further investigations were needed to ascertain mechanism. The clinicians should pay more attention to identify potential victims of neurologic complications, to reduce the mortality rate of snake bite.

## 1 Introduction

Snakebites are reported to have very high mortality, with an annual mortality rate up to 421,000 envenomings and 20,000.^[1]^ It leads to acute cerebral infarction, which is a serious problem for physicians who lack the necessary experience in diagnosing and treating snakebites. Mosquera et al^[2]^ analyzed the clinical data of 309 patients with snakebites and found that only 8 patients had cerebrovascular complications, including 7 patients with cerebral hemorrhage and only 1 patient with cerebral infarction.

This study aimed to present the case of a 49-year-old woman who was bitten by a *Trimeresurus stejnegeri*. On the fourth day after the bite, she developed nonfluent aphasia with difficulty in expression and understanding. She also had an acute onset of right hemiplegia. This case study indicated that clinicians should pay attention to acute cerebral infarction in relation to snakebites.

## 2 Case presentation

**Figure 1:**
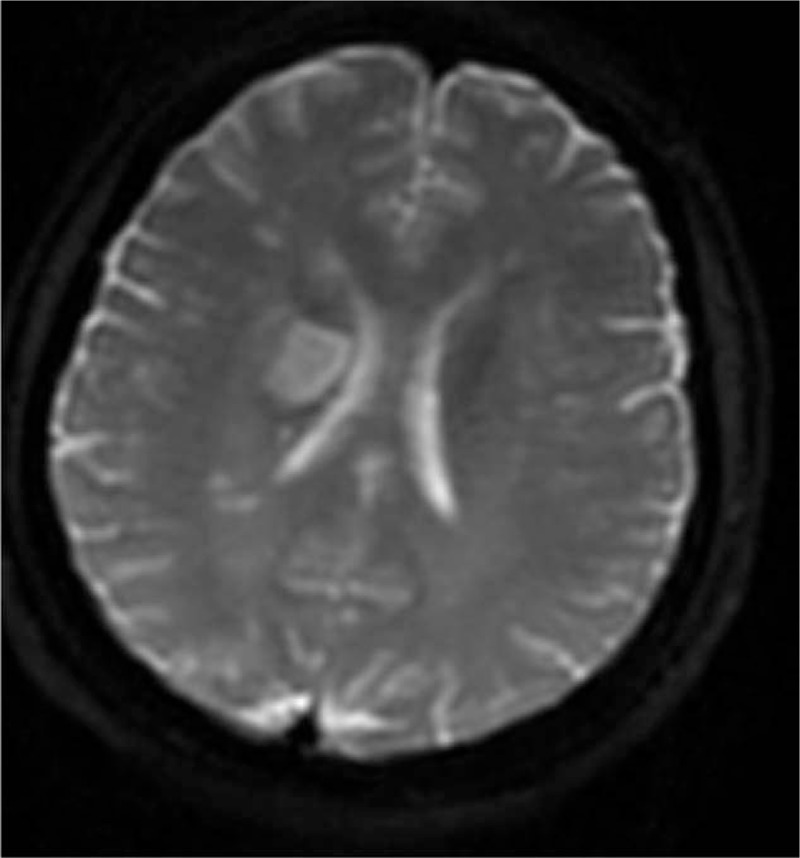
Magnetic resonance image of the patient.

A 49-year-old woman had a snakebite on her left foot while walking on the street. The killed snake was identified as *T. stejnegeri*. The patient developed severe pain and swelling in the left foot, local erythema, and ecchymosis a few minutes after the bite. She was taken to a nearby clinic where she was given base treatment, including cleaning the wound and hemostasis. For further treatment, she was subsequently transferred to the hospital. On examination, the vital signs were found to be stable and the left foot was markedly swollen. The neurological examination was essentially normal on admission. She was immediately treated with three 10-mL intravenous injections of polyvalent anti-snake venom serum. Meanwhile, she was also injected with adsorbed tetanus toxoid. She also received ceftriaxone and other supportive therapy. The laboratory findings were as follows: mild leukocytosis and negative coagulation function and fibrin degradation products. The patient developed right-side weakness and speech disturbances on the fourth day after the bite. She also had nonfluent aphasia with difficulty in expression and understanding and right spastic hemiparesis involving the face, arm, and, to a lesser degree, the leg. The examination revealed that edema in the left lower extremity was obvious. The nervous system examination showed that the consciousness was clear. The patient had mixed aphasia. The bilaterally round pupils, about 3 mm in diameter, were sensitive to light reflection. Further, the nasolabial fold was shallow on the right, the tongue deviated to the right, and the right limb muscle strength was 0. The left limb muscle strength was of grade 5, the Babinski sign on the right side was positive, and the National Institutes of Health Stroke Scale (NIHSS) score was 18 points. The brain computed tomography scan showed no cerebral hemorrhage symptoms. Magnetic resonance imaging showed acute ischemic infarct in the left territory (Fig. 1). Magnetic resonance angiography of the cerebral circulation revealed no abnormalities. Electrocardiogram demonstrated sinus tachycardia. The color Doppler study showed no arterial or venous thrombosis in the lower limbs. The workup for the other stroke risk profiles, including lipoprotein (a), serum homocysteine, and antithrombin III; carotid Doppler; and 2-dimensional echocardiography was normal.

The patient was treated with neuroprotective therapy (edaravone 30 mg, once a day) for 10 days and antiplatelet aggregation (clopidogrel 75 mg, once a day). Also, polyvalent anti-snake venom serum was injected. Two weeks later, the swelling in the left lower extremity of the patient disappeared, and the right limb muscle strength recovered to grade 3. The patient had mixed aphasia, and the NIHSS score was 13 points. The myocardial injury markers and coagulation indexes were all within the reference range. The patient was discharged. After discharge, the patient continued to take clopidogrel for 3 months. The follow-up showed that the modified Rankin scale score was 4 points after 3 months.

### 2.1 Ethical approval

Ethical approval was not necessary because our study was a case report.

### 2.2 Patient approval

The patient signed informed consent for publication of the case.

## 3 Discussion

In this study, the snakebite resulted in infarction in the left territory of the patient. The clinical and biological progressions were favorable. This was the first case in which *T. stejnegeri* bite led to infarction. The incidence of infarction despite treatment for 4 days after the bite was surprising, suggesting that antivenom treatment might have been ineffective in this patient.

The number of case reports on acute cerebral infarction and snakebites in the Chinese Biomedical Literature Database and PubMed between 1994 and September 2018 was 13,^[2–13]^ including 8 male and 6 female patients, with an age range of 5 to 62 years and a median of 45 years. Most of these cases were reported in Asia and South America. The symptoms developed 2 hours after the snakebite, peaking after 4 hours. Infarcts were found in the front cycle or post-loop, but did not simultaneously happen on both circulatory. The prognosis of adolescents and children was poor (Table 1). In conclusion, a venomous snakebite caused cerebral infarction with high mortality and poor prognosis. This may be because, besides systemic multiple-organ damage, the cerebral collateral circulation did not have sufficient opening time when the acute cerebral infarction occurred, the collateral vascular compensation was poor, and the systemic blood was in a hypercoagulable state, resulting in a large cerebral infarction area after the snakebite. Moreover, the prognosis was poor and the mortality rate was high.

**Table 1:**
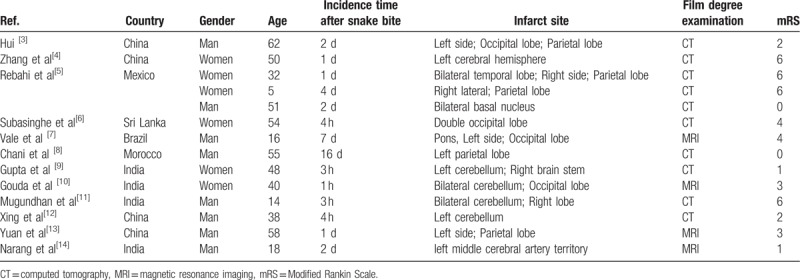
General information and prognosis of patients with cerebral infarction caused by poisonous snake bites from 1994 to September 2018.

*T. stejnegeri* inhabits wet areas. It was first found in India. The venom of this snake is a complicated mixture of several enzymes and proteins, toxic polypeptides, and inorganic components. It contains more than 1 toxin, and their combined effect is more potent than their individual effects. It is mainly described as hemotoxic. The main components of venom are as follows ^[14]^: first, proteolytic enzymes, which catalyze the breakdown of tissue structural proteins and lead to pain and swelling at the site of the bite; second, polypeptide toxins (e.g., viper toxin), which disrupt the nerve-impulse transmission and may lead to heart or respiratory failure; third, proteases, which disrupt protein–peptide bonds in tissues, resulting in damage to the wall of blood vessels, hemorrhage, and muscle fiber destruction; fourth, phospholipases, which catalyze reactions that harm nerves and muscles; fifth, collagenases, which lead to the destruction of connective tissue collagen; and sixth, thrombin-like enzymes, which interfere with normal blood clotting. The manifestations following snakebites depend on the severity of envenomation. In cases of minimal envenomation, only local signs at the bite site are observed, mainly including swelling, erythema, and ecchymosis. Systemic manifestations are either insignificant or absent. In cases of moderate envenomation, the local signs may also include the presence of blisters and involve a larger part of the affected location. Systemic symptoms may be present, but they are not life-threatening (mild hypotension, tachycardia, and tachypnea). In cases of severe envenomation, the local signs are profound, involving the entire affected location. They spread rapidly and include hemorrhagic edema and tissue necrosis. In this study, snake envenomation caused other complications (neurologic signs and symptoms). The patient had hypofibrinogenemia because of the coagulating effect of the venom. Meanwhile, the venom caused arrhythmias (sinus tachycardia).

*T. stejnegeri* snakebites exhibit both anticoagulant and coagulant effects.^[15]^ The coagulant effect may be a result of arginine esterase hydrolase, an enzyme that is similar in action to thrombin. It clots fibrinogen and aggregates platelets. These coagulant effects may also be due to the conversion of prothrombin into thrombin, a change catalyzed by proteinases.^[15]^ This triggering of the coagulation cascade in vivo results in the formation of microthrombi, fibrinolysis, and a bleeding tendency, leading to hemorrhagic complications.^[14,16]^

However, acute cerebral infarction after *T. stejnegeri* bites is rare. The putative mechanism causing the thrombotic process and cerebral infarcts remains complex and unknown. They occur 36 hours after the snakebite (ranging from 7 hours to 4 days).^[17]^ The bite can cause massive disseminated intravascular coagulopathy with vessel occlusions, resulting in cerebral infarction. The procoagulant activity of the venom, vascular damage, and hyperviscosity secondary to circulatory shock may also contribute to cerebral infarcts. In patients with a snakebite, vascular endothelial damage is usually caused by venom hemorrhagins or metalloproteinases.^[18–20]^ The most significant histopathological finding is the association of the infarctions with unusual and multifocal diffuse thrombotic microangiopathy involving small arteries and arterioles of major organs.^[4]^

At present, reports on cerebral infarction caused by snakebites are scarce. Hence, evidence-based guidance on the treatment plan is lacking. No other special treatment plan except the early detection of abnormal blood coagulation and symptomatic treatment is available. The findings of the recent emergency cerebrovascular thrombectomy study published in the *New England Journal* brought relief to such patients.^[21,22]^ Similar to the present case, early antiplatelet and neuroprotective therapies may be effective for patients with snakebites accompanied by cerebral infarction. Thrombolytic drugs and thrombectomy devices need to be developed for patients with thrombosis caused by snakebites, which is another worthwhile direction to reduce the mortality and morbidity caused by snakebites.

In conclusion, the uncommon cerebrovascular accidents in the present case were most likely due to generalized prothrombotic action of the venom and toxin-induced vasculitis instead of an underlying prothrombotic tendency. The timing of the stroke in relation to the bite needs further exploration. Detailed studies on envenomation in humans may improve the understanding of the pathogenesis of other vasculopathies and help in the identification of potential victims of neurological complications, ultimately leading to more effective treatment of these bites.

## Author contributions

**Data curation:** Mei Yan.

**Investigation:** Jin Lun Hu.

**Methodology:** Yi Xia Wu.

**Supervision:** Xiao Huan Liang.

**Validation:** Yue Fu.

**Visualization:** Meng Huan Zhu.

**Writing – original draft:** Xiang Xia Zeng.

Yue Fu orcid: 0000-0002-6848-2371.
